# Left ventricular ejection fraction and myocardial fibrosis in sudden cardiac death

**DOI:** 10.1093/europace/euaf306

**Published:** 2025-11-28

**Authors:** Harri Silvola, Lauri Holmström, Lasse Pakanen, Ida King, Anette Eskuri, Jani Tikkanen, Juha Perkiömäki, Heikki Huikuri, Juhani Junttila

**Affiliations:** Research Unit of Biomedicine and Internal Medicine, Medical Research Center Oulu, University of Oulu, Oulu University Hospital, OYS Sydän, Kajaanintie 50, Oulu 90220, Finland; Research Unit of Biomedicine and Internal Medicine, Medical Research Center Oulu, University of Oulu, Oulu University Hospital, OYS Sydän, Kajaanintie 50, Oulu 90220, Finland; Forensic Medicine Unit, Finnish Institute for Health and Welfare, Oulu, Finland; Department of Forensic Medicine, Research Unit of Biomedicine and Internal Medicine, Medical Research Center Oulu, University of Oulu, Oulu, Finland; Research Unit of Biomedicine and Internal Medicine, Medical Research Center Oulu, University of Oulu, Oulu University Hospital, OYS Sydän, Kajaanintie 50, Oulu 90220, Finland; Research Unit of Biomedicine and Internal Medicine, Medical Research Center Oulu, University of Oulu, Oulu University Hospital, OYS Sydän, Kajaanintie 50, Oulu 90220, Finland; Research Unit of Biomedicine and Internal Medicine, Medical Research Center Oulu, University of Oulu, Oulu University Hospital, OYS Sydän, Kajaanintie 50, Oulu 90220, Finland; Research Unit of Biomedicine and Internal Medicine, Medical Research Center Oulu, University of Oulu, Oulu University Hospital, OYS Sydän, Kajaanintie 50, Oulu 90220, Finland; Research Unit of Biomedicine and Internal Medicine, Medical Research Center Oulu, University of Oulu, Oulu University Hospital, OYS Sydän, Kajaanintie 50, Oulu 90220, Finland; Research Unit of Biomedicine and Internal Medicine, Medical Research Center Oulu, University of Oulu, Oulu University Hospital, OYS Sydän, Kajaanintie 50, Oulu 90220, Finland

**Keywords:** Sudden death, Cardiac arrest, Myocardial fibrosis, Echocardiography, Ejection fraction

## Abstract

**Aims:**

Left ventricular ejection fraction (LVEF) remains the key determinant in the evaluation for the risk of sudden cardiac death (SCD). Myocardial fibrosis has gained increasingly more interest in the context of various myocardial diseases. We determined the spectrum of LVEF and evaluated the association between myocardial fibrosis and pre-SCD LVEF in a population-based SCD cohort.

**Methods and results:**

The Fingesture study and clinical data have been collected from consecutive autopsy-verified SCD victims from Northern Finland between 1998 and 2017 (*n* = 5869). The cause of death was verified in medicolegal autopsy in all subjects. Electronic health records were used to identify those with pre-mortem echocardiography data. The extent of myocardial fibrosis at autopsy was characterized macroscopically and from histology samples. The LVEF recorded median 2 years (interquartile range 1–5) prior to SCD was evaluated in 716 SCD subjects. Proportional LVEF values were as follows: 62.7% (*n* = 449) normal LVEF (≥50%), 21.9% (*n* = 157) mildly reduced LVEF (36–49%), and 15.4% (*n* = 110) severely reduced LVEF (≤35%). At autopsy 19.6% (*n* = 140) had substantial, 53.8% (*n* = 386) moderate, and 22.1% (*n* = 158) mild fibrosis, and 4.5% (*n* = 32) had no myocardial fibrosis. The extent of myocardial fibrosis and LVEF had poor correlation (Spearman’s *ρ* 0.21, CI 0.141–0.285, *P* < 0.001). Only 21.4% of those with substantial fibrosis at autopsy had LVEF ≤35%.

**Conclusion:**

The proportion of SCD subjects with LVEF ≤35% is low, and the prevalence of myocardial fibrosis is high. The LVEF has a weak correlation with the extent of myocardial fibrosis. Our study suggests that LVEF is a poor surrogate of myocardial fibrosis in SCD victims.

## Graphical Abstract

**Graphical Abstract:**
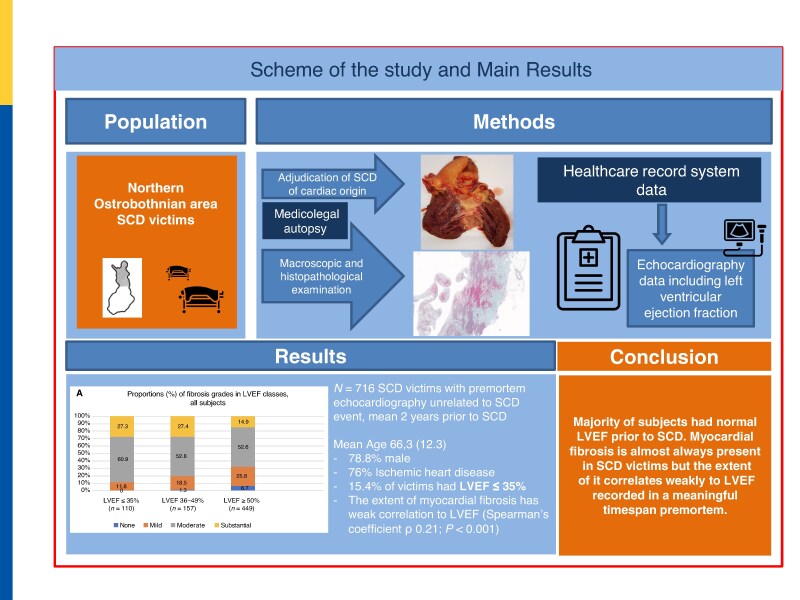

What’s new?

- Pre-mortem echocardiographies were analysed from a large population-based, autopsy-verified sudden cardiac death (SCD) cohort.
- Severely reduced LVEF (≤35%) was found only in 15.4% of subjects preceding SCD.
- Myocardial fibrosis was found at autopsy in ∼95% of SCD subjects with echocardiography performed prior to death.
- There is a weak correlation between pre-mortem left ventricular ejection fraction (LVEF) and severity of myocardial fibrosis in SCD, which suggests poor detectability of a potential fatal arrhythmic fibrotic substrate by LVEF.

## Introduction

Despite the improvements in the treatment of cardiac diseases, sudden cardiac death (SCD) causes ∼700 000 premature deaths in the USA and Europe on a yearly basis.^1–3^ The most important cardiac disease associated with SCD is coronary artery disease (CAD), comprising ∼70–80% of the cases, while non-ischaemic cardiac diseases account for ∼20–30% of SCDs in the general population.^4,5^ For decades, evaluation of SCD risk and patient selection for primary prevention implantable cardioverter-defibrillator (ICD) treatment have mainly been relying on the measurement of left ventricular ejection fraction (LVEF).^6–9^ Limitations of LVEF in predicting SCD have been identified, and alternative feasible strategies to identify individuals at risk are still needed.^10^

Several observational studies have found that excessive myocardial fibrosis is an important risk factor for SCD. It has been speculated that extent of myocardial fibrosis might have stronger association with SCD risk than reduced LVEF.^1,2^ In current clinical guidelines, myocardial fibrosis and scar burden assessed with cardiac magnetic resonance (CMR) have been added as a supportive finding in the evaluation of primary prevention ICD implantation in selected aetiologies like hypertrophic cardiomyopathy and dilated cardiomyopathy (DCM)^6,7^. As echocardiography remains the most widely available method of cardiac imaging, the accuracy of LVEF in the assessment of myocardial fibrosis among SCD victims needs clarification. Growing evidence on the importance of myocardial fibrosis across different aetiologies is unavoidably raising a question about its role in the overall SCD population, which has not been comprehensively studied to date. In this study, we utilized a large cohort of autopsy-verified SCD victims to investigate the prevalence of decreased LVEF in the SCD population and evaluate the association of LVEF with extent of myocardial fibrosis.

## Methods

### The Fingesture study

The study population is derived from the Finnish Genetic Study of Arrhythmic Events (Fingesture), which has gathered medical records and medicolegal autopsy data from 5869 consecutive unexpected SCD victims between 1998 and 2017 from the Oulu University Hospital District (defined geographical area in Northern Finland, population ≈600 000). The majority (56.4%) of subjects did not have any diagnosed cardiac disease prior to SCD. The detailed study protocol has been previously described, and a brief description follows.^11,12^

Finnish law requires a medicolegal autopsy to be performed if (i) the death is not due to a known disease, (ii) the victim has not been treated during his/her last illness, or (iii) the death is otherwise unexpected. Accordingly, all victims of sudden and unexpected death undergo meticulous post-mortem investigations in Finland. Because of this, Finland has the highest autopsy rate following sudden death in Western societies. All SCD victims in the Fingesture study underwent a medicolegal autopsy in the Finnish Institute for Health and Welfare, Oulu, Finland, or at the Department of Forensic Medicine, University of Oulu, Oulu, Finland. Autopsies were performed by experienced forensic pathologists, each performing over 100 autopsies per year, using a uniform study protocol and contemporary guidelines for diagnosing the cause of death. In sudden death subjects younger than 70 years old, the autopsy rate is 90.8% as has been previously reported.^13^

### Autopsy procedure

The Fingesture study included all victims of sudden death that were determined to be due to cardiac disease at autopsy. Non-cardiac causes (e.g. pulmonary embolism, aortic rupture, cerebrovascular event, trauma, and intoxication) were excluded from the Fingesture study. Determination of the cause of death was based on the combination of data in medical records, police reports, and autopsy data. The death was defined as ischaemic if there was evidence of CAD and a fresh intracoronary thrombus, plaque rupture or erosion, intraplaque haemorrhage, or critical coronary stenosis (>75%) in the major coronary vessel/branch at autopsy. Subjects were classified as non-ischaemic when the underlying cause of death was other than CAD, e.g. hypertensive cardiomyopathy, other cardiomyopathy, or valvular disease. The causes of death were documented based on the International Classification of Diseases 10th Revision classes. The causes of SCD in the Fingesture study have been reported previously.

Complete autopsy procedures included gross cardiac examinations, including visual and histological coronary artery examination in subjects with uncertain plaque characteristics, valve investigation, heart weight measurement, and determination of myocardial fibrosis based on macroscopic and histological investigation on tissue samples taken from the heart. Histological examination was performed in all subjects, and a toxicology investigation was performed when autopsy findings were insufficient to define a cause of death or if there was any suspicion of extensive alcohol intake or medication or drug use.

### Assessment of myocardial fibrosis

Evaluation of macroscopic focal myocardial scar was based on visual gross examination. In addition, quantification of myocardial fibrosis from histological samples was categorized into four groups: (i) no fibrosis, (ii) mild fibrosis, (iii) moderate fibrosis, and (iv) substantial fibrosis. The four-degree fibrosis classification is based on experienced forensic pathologists’ verbal statement of the amount of myocardial fibrosis in the heart from histological samples as previously described.^14^ The presence of myocardial scar (replacement fibrosis) was based on comprehensive macroscopic myocardial dissection and the presence of interstitial fibrosis on three to five histological tissue samples from different myocardial sites of the left ventricle. The main areas for three routine histological samples were the interventricular septum, left ventricle anterior wall, and lateral wall. More samples were taken from selected areas on forensic pathologists’ discretion if myocardial disease was suspected.

### Echocardiography

After the autopsy procedure and the determination of the cause of death, data on pre-SCD echocardiography recordings were obtained retrospectively from electronic medical records for each SCD subject. Echocardiographies were recorded prior to and unrelated to the SCD event. The exact date for the examination was known for all victims. If multiple echocardiographies were available, the most recent one was used. Median time from an echocardiography recording to SCD was 2 years [interquartile range (IQR) 1–5 years, 79.3% with ≤5 years]. The LVEF was analysed primarily by Simpson’s method for most of the victims and secondarily by visual evaluation of the examiner or by M-mode measurement. Pre-SCD LVEF was categorized into three classes: normal (LVEF ≥50%), mildly to moderately reduced (LVEF 36–49%), and severely reduced (LVEF ≤35%). This classification was based on the current guidelines for heart failure and SCD prevention.^1^

### Statistical methods

IBM SPSS Statistics 28.0 was used to perform statistical analyses. Continuous data are presented as mean values with SD. Between-group differences were analysed with the *χ*^2^-test and Student’s *t*-test/analysis of variance (ANOVA) test for categorical and continuous data, respectively. All reported *P*-values are two-sided, and values < 0.05 were considered significant. Correlation of class variables was evaluated by Spearman’s correlation coefficient (*ρ*) and 95% CI. Confounding factors were explored by performing Spearman’s correlation tests in multiple stratified analyses.

The study complies with the Declaration of Helsinki and was approved by the Ethics Committee of the Northern Ostrobothnia Hospital District. The Finnish Institute for Health and Welfare approved the review of post-mortem data and electronic health record (EHR) data by the investigators (document record number: THL/97/6.02.00/2024).

## Results

### Study subject characteristics

**Figure 1:**
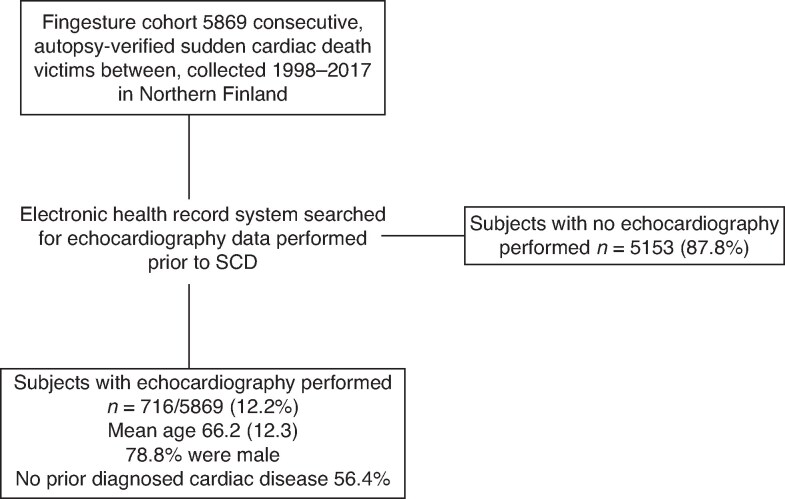
Study population derivation from the Fingesture study. Continuous data are presented as mean [SD]. Fingesture, Finnish Genetic Study of Arrhythmic Events; SCD, sudden cardiac death.

In total, we had pre-SCD echocardiography data with left ventricular function assessment in 716 SCD subjects derived from the Fingesture cohort (*n* = 5969) (*Figure 1*). The mean age of the study population was 66.2 (SD 12.3) years and 78.8% were male (*Table 1*). And 76% had ischaemic aetiology and 24% had non-ischaemic aetiology at autopsy. Subjects with pre-SCD echocardiography were slightly older [66.2 (12.3) vs. 64.7 (12.4) years; *P* = 0.003] compared to those with no previous echocardiography and had more often macroscopic myocardial scar and histological myocardial fibrosis. There were no differences in sex distribution or in the prevalence of ischaemic vs. non-ischaemic SCD.

**Table 1:** Demographic comparison between subjects with no echocardiography data available to those with available data

	Total *N* = 5869 (%)	LVEF not known *n* = 5153 (%)	LVEF known *n* = 716 (%)	*P*-value
Mean age (SD)	64.9 (12.4)	64.7 (12.4)	66.2 (12.3)	0.003
Female sex (%)	716 (12.2)	1086 (21.1)	152 (21.2)	0.92
Ischaemic SCD (%)	4392 (74.8)	3858 (74.7)	544 (76.0)	0.46
Heart weight at autopsy in grams (SD)	482 (128)	473 (123)	548 (144)	<0.001
Macroscopic myocardial scar	2411 (41.1)	1996 (38.7)	415 (58.0)	<0.001
Classification of myocardial fibrosis at autopsy (histological)^a^				<0.001
Substantial myocardial fibrosis	686 (11.7)	546 (10.6)	140 (19.6)	<0.05
Moderate myocardial fibrosis	2972 (50.6)	2586 (50.2)	386 (53.8)	NS
Mild myocardial fibrosis	1731 (29.5)	1573 (30.5)	158 (22.1)	<0.05
No fibrosis	480 (8.2)	448(8.7)	32 (4.5)	<0.05

LVEF, left ventricular ejection fraction; SCD, sudden cardiac death.

^a^Bonferroni correction for multiple comparisons was used.

### Echocardiography in sudden cardiac death victims

The majority of subjects with pre-SCD echocardiography had LVEF ≥50% (62.7%, *n* = 449/716), whereas 21.9% (157/716) had LVEF 36–49%, and 15.4% (110/716) had LVEF ≤35% (*Table 2*). Men were more likely to have reduced LVEF (<50%) than women (41.2% vs. 23.0%; *P* < 0.001). In addition, LVEF was found to be severely reduced (≤35%) more commonly in men compared to women (17.4% vs. 7.9%; *P* < 0.001). There was no significant difference in the mean age between LVEF categories, and ischaemic vs. non-ischaemic aetiology was equally predominant across all LVEF categories. In SCD victims with ischaemic and non-ischaemic heart disease, reduced LVEF (<50%) was equally common (37.7% vs. 36.0%, respectively; *P* = 0.72). The prevalence of severely reduced LVEF (≤35%) was also similar between ischaemic SCD and non-ischaemic SCD (15.1% vs. 16.3%, respectively; *P* = 0.72) (*Table 2*).

**Table 2:** Comparison according to LVEF classes

	LVEF ≤35%	LVEF 36–49%	LVEF ≥50%	*P*-value
Age (SD)	65.2 (11.3)	65.9 (12.4)	66.5 (12.4)	0.58
Female sex (%)	12 (10.9)	23 (14.6)	117 (26.1)	<0.001
Ischaemic SCD at autopsy (%)	82 (74.5)	123 (78.3)	339 (75.5)	0.72
Macroscopic myocardial scar	82 (74.5)	105 (66.9)	228 (50.8)	<0.001*
Total	110 (15.4)	157 (21.9)	449 (62.7)	

Bonferroni correction for multiple comparisons was used.

LVEF, left ventricular ejection fraction; SCD, sudden cardiac death.

^*^ *P*-value was significant between LVEF >50% and the two other groups and non-significant between LVEF >35 and LVEF 36–49%.

### Myocardial fibrosis

Myocardial scar at autopsy was present in 415 (57.7%) among all subjects, 36 (20.9%) among non-ischaemic SCD, and 379 (69.7%) among ischaemic SCD. The extent of myocardial fibrosis at autopsy was substantial in 19.6% (*n* = 140/716), moderate in 53.9% (*n* = 386/716), mild in 22.1% (*n* = 158/716), and non-detectable in 4.5% (*n* = 32/716) of SCD victims. Subjects with no fibrosis were younger compared to those with substantial fibrosis (61.0 and 67.3 years, respectively; *P* < 0.001). The proportion of female subjects was higher in those categories with less extensive myocardial fibrosis (*P* = 0.003). Ischaemic aetiology for SCD was proportionally more common when there was more extensive myocardial fibrosis at autopsy (*P* < 0.001) (*Table 3*).

**Table 3:** Demographic comparison according to extent of myocardial fibrosis

	No fibrosis *n* = 32	Mild fibrosis *n* = 158	Moderate fibrosis *n* = 386	Substantial fibrosis *n* = 140	*P*-value
Age (SD)	61.0 (14.4)	62.8 (12.9)	66.1 (11.6)	67.3 (11.6)	<0.001
Female sex (%)	14 (43.8)	37 (23.4)	81 (21.0)	20 (14.3)	0.003
Ischaemic SCD (%)	19 (59.4)	95 (60.1)	302 (78.2)	128 (91.4)	<0.001

SCD, sudden cardiac death.

### Left ventricular ejection fraction and myocardial fibrosis

**Figure 2:**
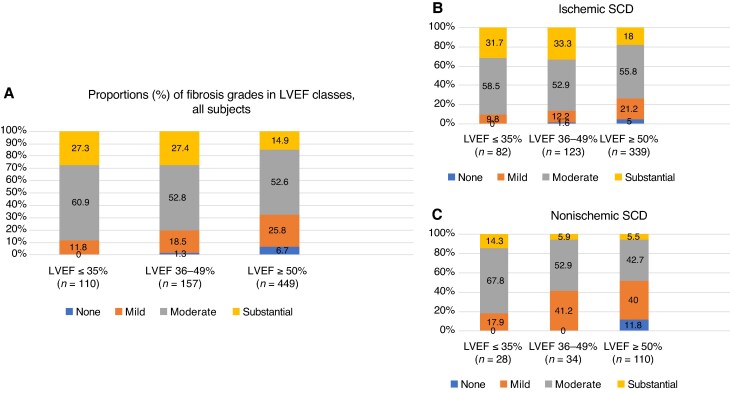
Severity of myocardial fibrosis according to LVEF. Moderate fibrosis and substantial fibrosis are common in all LVEF categories in both ischaemic and non-ischaemic SCD subjects. LVEF, left ventricular ejection fraction; SCD, sudden cardiac death.

**Figure 3:**
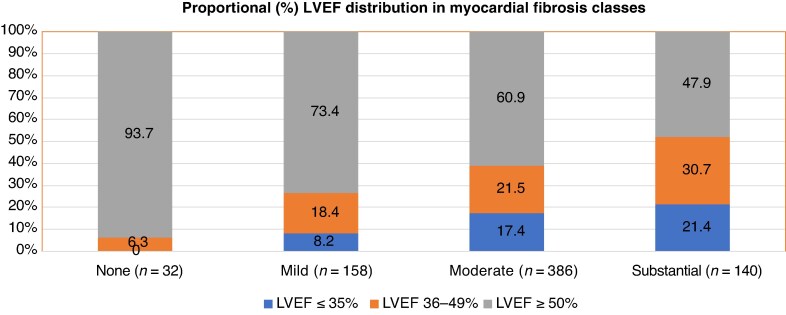
Spectrum of LVEF according to the extent of myocardial fibrosis. LVEF, left ventricular ejection fraction.

Those with LVEF ≤35% had more often substantial (27.3%) or moderate fibrosis (60.9%) compared to those with normal LVEF (15.0 and 52.5%, respectively) (*P* < 0.001) (*Figure 2*). Subjects with no detectable myocardial fibrosis at autopsy had normal LVEF in 93.7%. However, nearly half (47.9%, *n* = 67) of those with substantial fibrosis had normal LVEF and only 21.4% had LVEF ≤35%. Subjects with moderate myocardial fibrosis at autopsy had normal LVEF in 60.9% (*n* = 235), and LVEF was ≤35% in 17.4% (*n* = 67) (*Figure 3*). Sensitivity of LVEF ≤35% to detect substantial myocardial fibrosis was 21.4% (30/140) and specificity was 86.1% (495/575), and for substantial or moderate fibrosis, sensitivity was 18.4% (97/526) and specificity was 93.2% (177/190).

In ischaemic SCD with substantial myocardial fibrosis, LVEF was ≤35% in 20.3% (*n* = 26) and normal in 47.7% (*n* = 61). In ischaemic subjects with moderate myocardial fibrosis, LVEF was normal in 62.6% (*n* = 189). In non-ischaemic subjects, normal LVEF was observed prior to SCD in 50.0, 56.0, and 69.8% with substantial, moderate, and mild myocardial fibrosis, respectively (*Figure 2*). Details of the relation of LVEF and myocardial fibrosis are presented in the supplementary material for ischemic and nonischemic SCD and separately for both sexes (see Supplementary material online, *Tables S1–S4* respectively).

There was poor correlation between the extent of myocardial fibrosis and LVEF (Spearman’s *ρ* 0.21, CI 0.141–0.285, *P* < 0.001). We performed sensitivity analysis for those with more recent (≤1 and ≤2 years) echocardiography prior to SCD and found that the correlation remained poor (see Supplementary material online, *Table S5*). The correlation remained poor also in stratified analysis based on age, sex, and ischaemic aetiology (see Supplementary material online, *Table S5*).

## Discussion

In this large SCD study, we aimed to investigate the spectrum of LVEF and association between LVEF and myocardial fibrosis. We found that severely reduced LVEF precedes SCD in only ∼15% of cases, which is much lower than previously reported two decades earlier.^15,16^ Stecker *et al.* found a 30% proportion of subjects with severely reduced LVEF in OR, USA, and de Vreede-Swagemakers *et al*. found that 26% of those with known cardiac history had previous heart failure in Maastricht, Netherlands. This difference may be the result of enhanced prevention and treatment of heart failure with modern therapies in both ischaemic and non-ischaemic cardiac diseases over the decades. We estimate that the actual proportion of those with severely reduced LVEF is much rarer in the whole SCD population assuming that it is less likely to be present in those without the need for pre-SCD echocardiography.

Our data demonstrate that myocardial fibrosis is a common finding in SCD victims. As expected, myocardial fibrosis was pronounced in those with severely reduced LVEF. The LVEF as a measurement of function rather than characterization of the tissue to detect myocardial fibrosis is known to have limitations.^17^ In the present study, we showed that the large proportion of SCD subjects with moderate or substantial fibrosis did not have severely reduced LVEF.

Moderate or substantial myocardial fibrosis was commonly present in all LVEF categories. Therefore, quantification of LVEF is a largely insufficient measurement to detect myocardial fibrosis. The CMR imaging enables characterization of the myocardial tissue and quantification of fibrosis more accurately than echocardiography and may provide additional value in SCD risk stratification.^17^ Extensive late gadolinium enhancement (LGE) detected with cardiac MRI has been shown to improve identification of those patients otherwise considered to be at lower risk by two-fold.^18^ The type and quantity of significant myocardial scarring predisposing to SCD in different cardiomyopathies/clinical scenarios need more specific studies, but an ongoing clinical trial is already aiming to investigate the efficacy of primary prevention ICD among individuals with mild-to-moderate left ventricular systolic dysfunction and LGE in CMR.^18^ The SCD is often thought to occur as a result of an acute myocardial infarction, but we have previously reported that the majority of SCDs in CAD patients do not associate with acute plaque complications at autopsy but instead 96% have fibrosis or hypertrophy.^19^ The role of CMR in determining scar burden in SCD prevention needs further evaluation in individuals with CAD and LVEF above 35%.

The LVEF and myocardial fibrosis have poor correlation, and most of the subjects with moderate and nearly half of the subjects with substantial myocardial fibrosis had normal LV function. Severely reduced LVEF has poor sensitivity at detecting subjects with accumulation of myocardial fibrosis and at increased risk for SCD. Our study demonstrates that myocardial fibrosis, which may act as a potentially arrhythmogenic substrate for fatal arrhythmias, is poorly captured by LVEF measurement in the SCD population. There is clearly a need for alternative strategies to identify myocardial fibrotic accumulation not only in selected cardiomyopathy populations but also at the population level. Electrocardiography (ECG)-based phased triage with combined artificial intelligence analysis models could provide more accuracy in identifying subjects at risk for SCD and guide selection to substrate imaging such as CMR in the future.

As acknowledged in previous studies and demonstrated by the present study, the need to perform autopsies systematically cannot be highlighted enough.^20^ Especially in younger (<50 years old) SCD subjects, potentially inherited non-ischaemic cardiomyopathies are a common cause.^21,22^ These cardiomyopathies often characterized by myocardial fibrosis may not be detected in examinations performed during lifetime, and therefore, post-mortem diagnosis could potentially affect the prognosis of the relatives by early identification of the disease.

### Clinical implications

The present data demonstrate that targeting preventive measures to the population with LVEF ≤35% is truly only catching the tip of the iceberg, especially in the modern era, and at the individual level, identifying myocardial fibrosis predisposing to SCD may be more important than previously thought. Identifying fibrotic cardiomyopathy at the population level requires feasible and cost-effective methods for large-scale screening. An opportunistic two-step screening method might be the most feasible approach as a simple 12-lead ECG can help to select subjects for further cardiac examination.^23^

Prior prospective cohort studies have developed SCD risk prediction models that have identified variables in addition to LVEF that improve risk stratification,^24–26^ and a previously published multivariable risk model was developed to specifically predict SCD with shockable initial rhythm.^27^ Such models have shown improved SCD risk stratification over LVEF measurement but are limited by the requirement of comprehensive clinical examinations, which are often deficient among SCD victims in the general population. Although the vast majority of subjects have evidence of CAD or acquired structural cardiac disease at autopsy, SCD is often the first manifestation of the underlying cardiac disease, and only about half of the subjects have been diagnosed with a cardiac disease prior to their death.^28^ Therefore, improvements in the public health and efficient management of cardiovascular risk factors may have the highest impact on reducing SCD. The Lancet Commission on SCD recently placed substantial emphasis on the need to develop a multidisciplinary strategy for SCD prevention.^3^ A large global meta-analysis recently demonstrated that both incident cardiovascular disease and mortality related to it seem to be largely attributable to five modifiable risk factors: high body mass index, elevated systolic blood pressure, elevated non-HDL cholesterol, current smoking, and diabetes.^29^

### Strengths and limitations

Previous smaller-scale studies have studied associations of left ventricular functional measurements and myocardial fibrosis in selected populations.^30–34^ To our knowledge, this is the first study to examine the relationship between LVEF and myocardial fibrosis in SCD victims in a representative large SCD cohort. The following unique aspects made this study feasible. The Fingesture is a large study of autopsy-verified unexpected SCD victims, and all SCD victims are consecutively collected throughout the whole data collection period. The basis of this data collection is Finnish law, which requires a medicolegal autopsy including macroscopic and histopathologic evaluation of the myocardium to be performed in case of sudden death, and this law has remained unchanged for the relevant parts during the study period enabling a truly representative cohort from Northern Finland.^13^ Pre-SCD echocardiographies were obtained from electronic medical records and were performed in a clinically meaningful time span prior to and unrelated to the SCD event (median time 2 years, 79.3% with ≤5 years). By definition, SCD is an unexpected event, and prior studies have found that ∼40–50% of all SCDs occur in the absence of prior cardiac disease diagnosis.^28^ Hence, most of the victims do not have previous echocardiography.

This study has limitations regarding the representability of the whole SCD population as echocardiography was not available for the majority of SCD victims. The study population comprises subjects who had echocardiography performed for any reason prior to death creating a type of referral bias: those with echocardiography may have been more symptomatic and have better access to echocardiography for any reason compared to those without, which could lead to overestimation of severely reduced LVEF in the whole SCD population.

This study may not capture all those subjects evaluated for high risk of SCD who may have received life-saving therapy such as ICD, which may have resulted to a selection bias. Yet we believe this bias did not significantly affect the results as ICD was not an exclusion criterion to be included in this study.

Other data (left ventricle diameters, wall thickness, and other functional analysis) apart from LVEF were not reported consistently enough and subsequently were not included in the study analysis.

Although the autopsy procedure has remained standardized and was carried out by very experienced forensic pathologists over the two-decade study period, the amount of histological samples used to determine the amount of fibrosis is lower than modern recommendations and could lead to underestimation of myocardial fibrosis severity in some areas of the myocardium, which were not histologically sampled.^35,36^

In this study, we aimed to evaluate the correlation between LVEF and myocardial fibrosis at autopsy. By the current study design, we are not able to establish whether evaluation of myocardial fibrosis with any method is more valuable than LVEF in risk assessment for SCD, which remains a challenge for future research.

## Conclusions

The prevalence of severely reduced LVEF was low, and the extent of myocardial fibrosis was high among SCD victims. The association between reduced LVEF and myocardial fibrosis was weak, highlighting that myocardial fibrosis is a common denominator not only in specific cardiac diseases but also at the population level across different SCD phenotypes and LVEF categories. This study demonstrates the importance of identifying and characterizing myocardial fibrosis as a substrate instead of low LVEF in the evaluation of factors that may predispose to SCD as well as the importance of performing autopsies in sudden unexpected deaths especially in the younger population.

## Supplementary Material

euaf306_Supplementary_Data

## Data Availability

The data underlying this article will be shared on reasonable request to the corresponding author.
