# Supplementary material for: Left ventricular ejection fraction and myocardial fibrosis in sudden cardiac death
Source: Europace. 2025 Nov 28;27(12):euaf306. doi: 10.1093/europace/euaf306 (PMC12703369; doi:10.1093/europace/euaf306)
Supplement: euaf306_Supplementary_Data [file euaf306_supplementary_data.docx]

| **Supplementary material**  LVEF and myocardial fibrosis in ischemic, nonischemic, male and female subsets are presented respectively in tables 1-4.  Sensitivity analysis for different time delays from echocardiography to SCD and stratified analysis for the effect of possible confounding factors on correlation of LVEF and myocardial fibrosis are presented in table 5.  **Table 1. Ischemic SCD subjects** | | | | | | |
| --- | --- | --- | --- | --- | --- | --- |
|  | | | Left Ventricular ejection fraction classes and myocardial fibrosis at autopsy | | | Total |
|  |  |  | LVEF≤35% | EF36-49 | LVEF≥50% |  |
| fibrosis | Substantial | Count | 26_a_ | 41_a_ | 61_b_ | 128 |
|  |  | % within fibrosis | 20,3% | 32,0% | 47,7% | 100,0% |
|  |  | % within LVEF | 31,7% | 33,3% | 18,0% | 23,6% |
|  |  | % of Total | 4,8% | 7,6% | 11,2% | 23,6% |
|  | Moderate | Count | 48_a_ | 65_a_ | 189_a_ | 302 |
|  |  | % within fibrosis | 15,9% | 21,5% | 62,6% | 100,0% |
|  |  | % within LVEF | 58,5% | 52,9% | 55,8% | 55,4% |
|  |  | % of Total | 8,8% | 12,0% | 34,6% | 55,4% |
|  | Mild | Count | 8_a_ | 15_a_ | 72_a_ | 95 |
|  |  | % within fibrosis | 8,4% | 15,8% | 75,8% | 100,0% |
|  |  | % within LVEF | 9,8% | 12,2% | 21,2% | 17,5% |
|  |  | % of Total | 1,5% | 2,8% | 13,3% | 17,5% |
|  | None | Count | 0_a_ | 2_a_ | 17_a_ | 19 |
|  |  | % within fibrosis | 0,0% | 10,5% | 89,5% | 100,0% |
|  |  | % within LVEF | 0,0% | 1,6% | 5,0% | 3,5% |
|  |  | % of Total | 0,0% | 0,4% | 3,1% | 3,5% |
| Total | | Count | 82 | 123 | 339 | 544 |
|  |  | % within fibrosis | 15,1% | 22,6% | 62,3% | 100,0% |
|  |  | % within LVEF | 100,0% | 100,0% | 100,0% | 100,0% |
|  |  | % of Total | 15,1% | 22,6% | 62,3% | 100,0% |
| \| **Table 2. Nonischemic SCD subjects** \| \| \| \| \| \| \| \| --- \| --- \| --- \| --- \| --- \| --- \| --- \| \|  \| \| \| Left Ventricular ejection fraction classes and myocardial fibrosis at autopsy \| \| \| Total \| \| LVEF≤35% \| EF36-49 \| LVEF≥50% \| \| fibrosis \| Substantial \| Count \| 4_a_ \| 2_a_ \| 6_a_ \| 12 \| \| % within fibrosis \| 33,3% \| 16,7% \| 50,0% \| 100,0% \| \| % within LVEF \| 14,3% \| 5,9% \| 5,5% \| 7,0% \| \| % of Total \| 2,3% \| 1,2% \| 3,5% \| 7,0% \| \| Moderate \| Count \| 19_a_ \| 18_a_ \| 47_a_ \| 84 \| \| % within fibrosis \| 22,6% \| 21,4% \| 56,0% \| 100,0% \| \| % within LVEF \| 67,8% \| 52,9% \| 42,7% \| 48,8% \| \| % of Total \| 11,0% \| 10,5% \| 27,3% \| 48,8% \| \| Mild \| Count \| 5_a_ \| 14_a_ \| 44_a_ \| 63 \| \| % within fibrosis \| 7,9% \| 22,2% \| 69,8% \| 100,0% \| \| % within LVEF \| 17,9% \| 41,2% \| 40,0% \| 36,6% \| \| % of Total \| 2,9% \| 8,1% \| 25,6% \| 36,6% \| \| None \| Count \| 0_a_ \| 0_a_ \| 13_a_ \| 13 \| \| % within fibrosis \| 0,0% \| 0,0% \| 100,0% \| 100,0% \| \| % within LVEF \| 0,0% \| 0,0% \| 11,8% \| 7,6% \| \| % of Total \| 0,0% \| 0,0% \| 7,6% \| 7,6% \| \| Total \| \| Count \| 28 \| 34 \| 110 \| 172 \| \| % within fibrosis \| 16,3% \| 19,8% \| 64,0% \| 100,0% \| \| % within LVEF \| 100,0% \| 100,0% \| 100,0% \| 100,0% \| \| % of Total \| 16,3% \| 19,8% \| 64,0% \| 100,0% \|  \| **Table 3. Male SCD subjects** \| \| \| \| \| \| \| \| --- \| --- \| --- \| --- \| --- \| --- \| --- \| \|  \| \| \| Left Ventricular ejection fraction classes and myocardial fibrosis at autopsy \| \| \| Total \| \| LVEF≤35% \| EF36-49 \| LVEF≥50% \| \| fibrosis \| Substantial \| Count \| 27_a_ \| 39_a_ \| 54_b_ \| 120 \| \| % within fibrosis \| 22,5% \| 32,5% \| 45,0% \| 100,0% \| \| % within LVEF \| 27,6% \| 29,1% \| 16,3% \| 21,3% \| \| % of Total \| 4,8% \| 6,9% \| 9,6% \| 21,3% \| \| Moderate \| Count \| 60_a_ \| 70_a_ \| 175_a_ \| 305 \| \| % within fibrosis \| 19,7% \| 23,0% \| 57,3% \| 100,0% \| \| % within LVEF \| 61,2% \| 52,2% \| 52,6% \| 54,0% \| \| % of Total \| 10,7% \| 12,4% \| 30,9% \| 54,0% \| \| Mild \| Count \| 11_a_ \| 23_a, b_ \| 87_b_ \| 121 \| \| % within fibrosis \| 9,1% \| 19,0% \| 71,9% \| 100,0% \| \| % within LVEF \| 11,2% \| 17,2% \| 26,3% \| 21,5% \| \| % of Total \| 2,0% \| 4,1% \| 15,5% \| 21,5% \| \| None \| Count \| 0_a_ \| 2_a_ \| 16_a_ \| 18 \| \| % within fibrosis \| 0,0% \| 11,1% \| 88,9% \| 100,0% \| \| % within LVEF \| 0,0% \| 1,5% \| 4,8% \| 3,2% \| \| % of Total \| 0,0% \| 0,4% \| 2,8% \| 3,2% \| \| Total \| \| Count \| 98 \| 134 \| 332 \| 564 \| \| % within fibrosis \| 17,4% \| 23,8% \| 58,8% \| 100,0% \| \| % within LVEF \| 100,0% \| 100,0% \| 100,0% \| 100,0% \| \| % of Total \| 17,4% \| 23,8% \| 58,8% \| 100,0% \| | | | | | | |

| **Table 4. Female SCD subjects** | | | | | | |
| --- | --- | --- | --- | --- | --- | --- |
|  | | | Left Ventricular ejection fraction classes and myocardial fibrosis at autopsy | | | Total |
|  |  |  | LVEF≤35% | EF36-49 | LVEF≥50% |  |
| fibrosis | Substantial | Count | 3_a_ | 4_a_ | 13_a_ | 20 |
|  |  | % within fibrosis | 15,0% | 20,0% | 65,0% | 100,0% |
|  |  | % within LVEF | 25,0% | 17,4% | 11,1% | 13,2% |
|  |  | % of Total | 2,0% | 2,6% | 8,6% | 13,2% |
|  | Moderate | Count | 7_a_ | 13_a_ | 61_a_ | 81 |
|  |  | % within fibrosis | 8,6% | 16,0% | 75,3% | 100,0% |
|  |  | % within LVEF | 58,3% | 56,5% | 52,1% | 53,3% |
|  |  | % of Total | 4,6% | 8,6% | 40,1% | 53,3% |
|  | Mild | Count | 2_a_ | 6_a_ | 29_a_ | 37 |
|  |  | % within fibrosis | 5,4% | 16,2% | 78,4% | 100,0% |
|  |  | % within LVEF | 16,7% | 26,1% | 24,8% | 24,3% |
|  |  | % of Total | 1,3% | 3,9% | 19,1% | 24,3% |
|  | None | Count | 0_a_ | 0_a_ | 14_a_ | 14 |
|  |  | % within fibrosis | 0,0% | 0,0% | 100,0% | 100,0% |
|  |  | % within LVEF | 0,0% | 0,0% | 12,0% | 9,2% |
|  |  | % of Total | 0,0% | 0,0% | 9,2% | 9,2% |
| Total | | Count | 12 | 23 | 117 | 152 |
|  |  | % within fibrosis | 7,9% | 15,1% | 77,0% | 100,0% |
|  |  | % within LVEF | 100,0% | 100,0% | 100,0% | 100,0% |
|  |  | % of Total | 7,9% | 15,1% | 77,0% | 100,0% |

Table 5. Correlation of LVEF and myocardial fibrosis- effect of confounding factors

|  | Spearmans ƿ | Confidence interval | P-value |  |
| --- | --- | --- | --- | --- |
| All study SCD subjects | 0.214 | 0.141 - 0.285 | <0.001 |  |
| Echocardiography ≤2 years prior to SCD | 0.199 | 0.101 -0.294 | <0.001 |  |
| Echocardiography ≤1 year prior to SCD | 0.225 | 0.114-0.331 | <0.001 |  |
| Sex |  |  |  |  |
| Men | 0.206 | 0.123-0.286 | <0.001 |  |
| Women | 0.171 | 0.007-0.326 | 0.035 |  |
| Age |  |  |  |  |
| < 67 years | 0.242 | 0.139-0.341 | <0.001 |  |
| ≥ 67years | 0.188 | 0.083-0.288 | <0.001 |  |
| Ischemic SCD at autopsy |  |  |  |  |
| Ischemic SCD | 0.209 | 0.125-0.290 | <0.001 |  |
| Nonischemic SCD | 0.259 | 0.109-0.397 | <0.001 |  |
| Macroscopic scar at autopsy |  |  |  |  |
| Scar | 0.118 | 0.019-0.215 | 0.016 |  |
| No scar | 0.117 | 0.061-0.287 | 0.002 |  |
